# Is it time to consider how to maximize the impact of simple epidemiological studies in the field of movement disorders?

**DOI:** 10.1055/s-0046-1827824

**Published:** 2026-10-09

**Authors:** Vitor Tumas, Bruno Lopes Santos-Lobato

**Affiliations:** 1 Universidade de São Paulo, Faculdade de Medicina de Ribeirão Preto, Departamento de Neurociências e Ciências do Comportamento, Ribeirão Preto SP, Brazil; 2 Hospital Ophir Loyola, Serviço de Neurologia, Belém PA, Brazil; 3 Universidade Federal do Pará, Laboratório de Neuropatologia Experimental, Belém PA, Brazil

The epidemiology of various movement disorders is influenced by the genetic characteristics and environmental exposure of populations in different regions of the world. Demographic factors, such as gender distribution and the age structure of the population, are also important contributing factors in determining the prevalence of movement disorders in a given population. Accurately understanding the distribution of these diseases is a fundamental step toward comprehending the factors associated with their origin in a given population and enables the planning of policies for their control, prevention, and mitigation of their impact. There is a lack of precise data on the epidemiology of movement disorders in Latin America, and studies providing this information are therefore very welcome.

Given the demographic, ethnic, and environmental diversity of this region, it is to be expected that there will be significant epidemiological differences in movement disorders compared with other regions of the world. Furthermore, there are certainly important differences among the various regions of Latin America itself. 1 Several studies have already pointed to these possible regional differences; however, the existing data must be analyzed very carefully, since most of these studies used very different methodologies, making it difficult to draw precise comparisons with other local studies and those from other parts of the world. The design and methodology used in epidemiological studies are crucial for determining the level of scientific evidence supporting the results and, ultimately, the relevance of the recommendations derived from such studies. 2 Therefore, there remains a significant lack of high-quality epidemiological data on movement disorders in the Latin American population.

In this issue of the Arquivos de Neuro-Psiquiatria, Farias-Moreno et al. presented the results of a retrospective analysis of clinical consultations conducted over a decade at a movement disorders specialized clinic in Guadalajara, Mexico. 3 The study's methodology is well described and elaborated, and the authors presented the results of an analysis of the medical records of 1,830 patients treated between 2012 and 2022. This is a descriptive, observational epidemiological study with a cross-sectional analysis of a sample from a specialized care center. Therefore, the sample in this study does not accurately reflect the distribution of movement disorders in that population and is subject to various potential biases. This type of study does not allow for the precise establishment of cause-and-effect relationships. Still, it can serve to identify potential associations that need to be confirmed in the future. However, it is a type of study that can be useful, especially for health services planning. Epidemiological studies of this type are the most common, despite their limitations, because they are low-cost and easy to conduct.

The authors showed that parkinsonism, tremors, and dystonia were the most common movement disorders, as observed in most similar specialized outpatient clinics. 4 They also found that parkinsonism was more common among older adults and males, as expected. They described associations between a diagnosis of parkinsonism and the presence of hypertension and smoking, and between hyperkinesias and a family history of similar disorders and illicit drug use. Approximately two-thirds of the sample consisted of patients over 50 years of age, and the majority of these cases presented with parkinsonism. Thus, the authors suggested that patients in this age group seen in healthcare settings—and especially those with hypertension—should be evaluated for the presence of parkinsonian signs. Similarly, they suggested that screening for cardiovascular problems should always be performed in patients with parkinsonism. An interesting and little-known epidemiological finding in Latin American outpatient clinics was that 3.7% of cases corresponded to functional movement disorders. Finally, the authors concluded that the study's findings are important for planning local public health services. Patients referred to this university-affiliated specialized service were largely without access to private healthcare, and the study revealed the complexity of the cases treated at these public outpatient clinics, which requires the presence of specialists in the field. Mexico's health care system consists of a private system and a multifaceted public system, which is in the process of transitioning to a universal public health care system.

Although it has numerous limitations, as the authors rightly pointed out, the study draws significant attention to the urgent need for more consistent regional epidemiological data on movement disorders in Latin America. Simple, low-cost epidemiological studies such as this one can play a major role in this process, but to do so, they must be based on well-defined hypotheses and objectives. For example, studies can be designed to investigate associations with previously suspected potential causal factors. In addition to determining the prevalence of a given disease in a population, we need to understand better specific genetic and environmental factors that may play a relevant role in each region. Future studies could compare the epidemiology of similar services across different regions, which could yield interesting insights. Studies could also be designed to analyze aspects of the organization of local health services. For example, these analyses could better define the process and the time elapsed until patients are referred to and treated at a specialized outpatient clinic within a given health system. In this way, such studies could provide more consistent and accurate data on the characteristics and care process for patients with movement disorders in Latin America.

While retrospective study designs are the simplest and most commonly used, there is no reason not to plan low-cost longitudinal studies, which can provide valuable information on the epidemiology of movement disorders in different regions of Latin America. Furthermore, it would be very important to conduct multicenter collaborative studies using more standardized methods. Perhaps now is the time for experts and authorities in the field to develop general recommendations for conducting low-cost epidemiological studies in regions of the world that lack resources and accurate data.
